# Specific Deoxyceramide Species Correlate with Expression of Macular Telangiectasia Type 2 (MacTel2) in a SPTLC2 Carrier HSAN1 Family

**DOI:** 10.3390/genes14040931

**Published:** 2023-04-18

**Authors:** Lindsey M. Q. Wilson, Sadaf Saba, Jun Li, Lev Prasov, Jason M. L. Miller

**Affiliations:** 1 Department of Human Genetics, University of Michigan Medical School, Ann Arbor, MI 48109, USA; 2 Center for Molecular Medicine and Genetics, Wayne State University School of Medicine, Detroit, MI 48201, USA; 3 Department of Neurology, Wayne State University School of Medicine, Detroit, MI 48201, USA; 4 Department of Ophthalmology and Visual Sciences, Kellogg Eye Center, University of Michigan Medical School, Ann Arbor, MI 48105, USA; 5 Cellular and Molecular Biology Program, University of Michigan Medical School, Ann Arbor, MI 48109, USA

**Keywords:** macular telangiectasia type 2 (MacTel2), serine palmitoyltransferase complex 1 (SPTLC1), serine palmitoyltransferase complex 2 (SPTLC2), hereditary sensory and autonomic neuropathy type 1 (HSAN1), ceramides, sphingolipids, deoxyceramides

## Abstract

Hereditary sensory and autonomic neuropathy type 1 (HSAN1/HSN1) is a peripheral neuropathy most commonly associated with pathogenic variants in the *serine palmitoyltransferase complex (SPTLC1, SPTLC2)* genes, which are responsible for sphingolipid biosynthesis. Recent reports have shown that some HSAN1 patients also develop macular telangiectasia type 2 (MacTel2), a retinal neurodegeneration with an enigmatic pathogenesis and complex heritability. Here, we report a novel association of a *SPTLC2* c.529A>G p.(Asn177Asp) variant with MacTel2 in a single member of a family that otherwise has multiple members afflicted with HSAN1. We provide correlative data to suggest that the variable penetrance of the HSAN1/MacTel2-overlap phenotype in the proband may be explained by levels of certain deoxyceramide species, which are aberrant intermediates of sphingolipid metabolism. We provide detailed retinal imaging of the proband and his HSAN1+/MacTel2- brothers and suggest mechanisms by which deoxyceramide levels may induce retinal degeneration. This is the first report of HSAN1 vs. HSAN1/MacTel2 overlap patients to comprehensively profile sphingolipid intermediates. The biochemical data here may help shed light on the pathoetiology and molecular mechanisms of MacTel2.

## 1. Introduction

**Figure 1:**
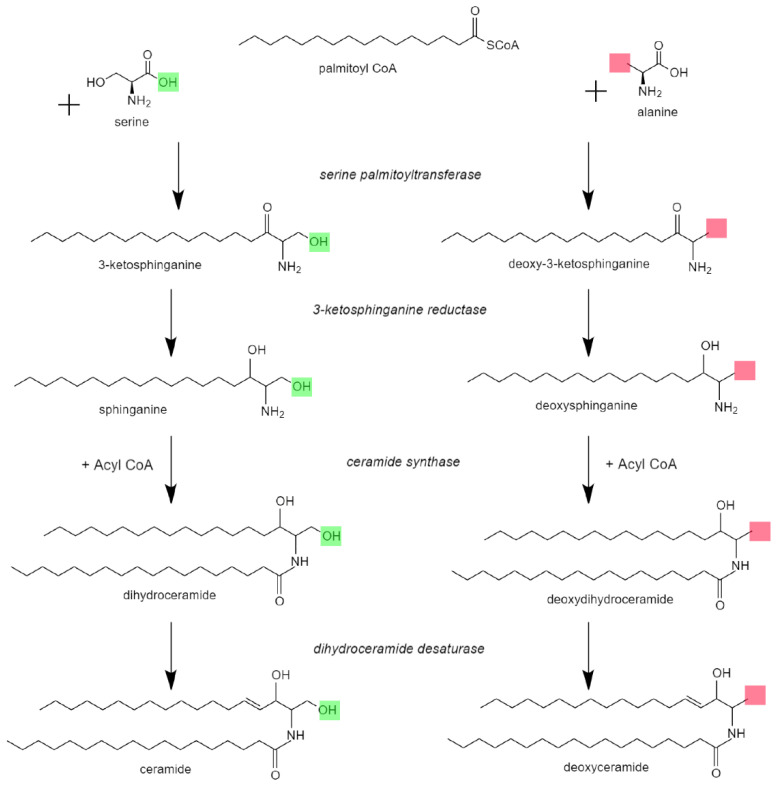
De novo sphingolipid biosynthesis pathway. The serine palmitoyltransferase (SPT) complex incorporates serine into palmitoyl-CoA as the first and rate-limiting step of sphingolipid biosynthesis. Alternatively, alanine (or glycine) can be incorporated in certain settings to produce deoxysphingolipids and deoxyceramides. If glycine is incorporated (not shown), deoxymethylsphingolipids are generated. The difference in products when incorporating alanine (or glycine) instead of serine is the lack of a hydroxyl group, highlighted in green where present and in red where absent.

**Figure 2:**
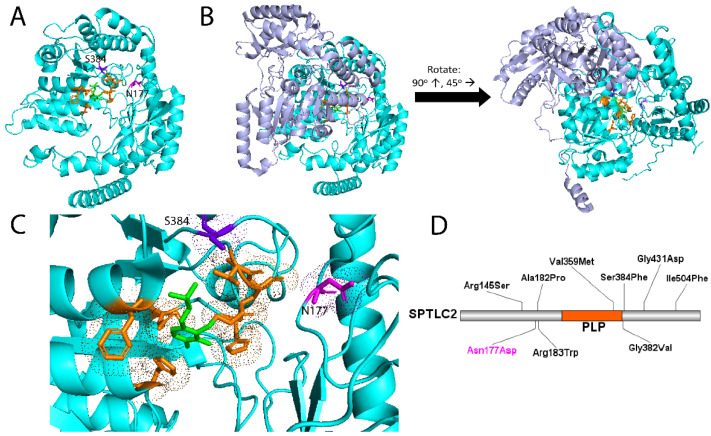
Serine palmitoyltransferase long-chain base subunit 2 (SPTLC2) molecular modeling and mutational analysis. (**A**) SPTLC2 protein (cyan) showing the proximity of the Asn177 residue (magenta) to the active site (orange). This residue is altered in the proband’s family. The only other mutation of SPTLC2 with HSAN1/MacTel2 overlap in the literature, located at Ser384, is notated for comparison (dark purple). PLP cofactor is green. SPTLC2 exists as a single isoform 562 residues in length [10]. (**B**) SPTLC2 (cyan) bound to serine palmitoyltransferase long-chain base subunit 1 (SPTLC1) (purple) to show that the active site (orange) is confined to the SPTLC2 subunit and not at the interface. The view is rotated upwards 90 degrees and left 45 degrees for better visualization of the active site. (**C**) Zoomed in view of (**A**), showing the electron density clouds of the residues involved in the active site (orange), PLP cofactor (green), and residues at the locations of Asn177 and Ser384, the known MacTel2-causing mutations (magenta and dark purple). (**D**) Locations of point mutations in SPTLC2 known to cause HSAN1. Highlighted in orange is the pyridoxal 5′-phosphate binding pocket (labeled “PLP”) and presumed active site. The proband family’s mutation is shown in magenta.

Hereditary sensory and autonomic neuropathy (HSAN) type 1 is classically characterized by peripheral paresthesia, pain, muscle cramps and weakness, sensorineural hearing loss, and intellectual disability. As many HSAN1 patients do not manifest with an autonomic neuropathy, the disease is also alternatively called hereditary sensory neuropathy (HSN1). HSAN1 is inherited in an autosomal dominant pattern with pathogenic variants in genes *SPTLC1*, *SPTLC2*, *RAB7A*, *ATL1*, and *DNMT1* [1]. Serine palmitoyltransferase long-chain base subunits 1 and 2 (SPTLC1 and SPTLC2) are two of the three components of the serine palmitoyltransferase (SPT) complex, which is responsible for converting palmitoyl-CoA and serine into 3-keto-sphinganine, the first and rate-limiting step in the de novo biosynthesis of sphingolipids (Figure 1) [2,3,4]. The SPT complex (480 kDa) has been well-characterized structurally and is formed by a dimer of two heterodimers; the heterodimers are formed by SPTLC1 (55 kDa) bound to SPTLC2 (65kDa) or STPLC3 [2,3] (Figure 2). SPT utilizes a pyridoxal 5′phosphate (PLP) co-factor to bind L-serine and allow for neutrophilic attack of the palmitoyl-CoA [5]. Only SPTLC2 and SPTLC3 have PLP binding sites and therefore form the enzymatically active domains [2,4]. Variants in SPT that are associated with HSAN1 are thought to disrupt the specificity of substrate binding (Figure 2). In particular, incorporation of alanine or glycine rather than serine in the SPT active site favors production of toxic deoxysphingolipids (Figure 1), leading to symptom onset [6,7,8,9].

Recently, some HSAN1 patients have been concurrently identified as having a retinal degenerative condition called macular telangiectasia type 2 (MacTel2). The molecular mechanisms and strength of association between HSAN1 and MacTel2 remain to be fully elucidated [11,12,13]. MacTel2 is a progressive retinal degeneration characterized by the loss of central, high-acuity vision. The primary cell type affected, Müller glia, is responsible both for helping maintain the blood–retinal barrier and providing trophic support for photoreceptors in the fovea, the central part of the retina responsible for high-acuity color vision. As Müller glia degenerate, both vascular and neurodegenerative consequences ensue, including vascular leakage and photoreceptor loss. Upon examination, Müller cell degeneration results in crystalline deposits within the retina [14].

Genetic analysis in sporadic MacTel2 has identified several candidate genes—*PSPH*, *PHGDH*, *CPS1*, and *ALDH1L1*—which all play a role in regulating serum serine levels [15,16,17]. For instance, PHGDH is the first step in serine biosynthesis [16,17]. Additionally, a metabolomics investigation of sporadic MacTel2 patients identified lipid dysregulation as a risk factor for the disease [18]. It is hypothesized that low levels of serine shift retinal sphingolipid and ceramide synthesis pathways (Figure 1) towards incorporation of alanine and production of toxic deoxysphingolipids, including deoxyceramide [11,15,19]. In turn, deoxysphingolipid accumulation may be particularly toxic to Müller glia, although this remains to be definitively proven.

Studies of HSAN1 families also support a link between serine metabolism and MacTel2. To date, there have been ten reported cases of patients with HSAN1 diagnosed with MacTel2, seven cases with the *SPTLC1* p.(Cys133Tyr) variant, and three with the *SPTLC2* p.(Ser384Phe) variant [11,13]. In these cases, high levels of deoxysphingolipids from failure of serine incorporation in the SPT complex were hypothesized to be the cause of MacTel2 development. However, another study involving thorough ocular examinations of 16 patients with HSAN1, including cases with the *SPTLC1* p.(Cys133Tyr) and *SPTLC2* p.(Ser384Phe) mutations, found that none of them had MacTel2 [12]. The reasons for this significantly reducing the penetrance of the HSAN1/MacTel2-overlap syndrome are unknown. One possibility that remains to be tested is that only patients with the highest levels of specific deoxysphingolipid species demonstrate MacTel2 symptoms.

Here, we provide a detailed analysis of a family with a *SPTLC2* p.(Asn177Asp) variant and HSAN1 [20], in which only the proband manifested with HSAN1/MacTel2 overlap. This variant has not previously been associated with MacTel2. We further provide high-resolution retinal imaging and comprehensive sphingolipid profiling of all family members, including those with HSAN1/MacTel2, with HSAN1 only, and with no disease. We find that the levels of certain deoxyceramide species correlate with the HSAN1/MacTel2-overlap phenotype, providing a hypothesis for variable penetrance and expressivity of MacTel2 in HSAN1 carriers and offering biochemical clues to the pathogenesis of sporadic MacTel2.

## 2. Materials and Methods

### 2.1. DNA Sequencing and Patient Consent

The proband underwent genetic testing through the Charcot–Marie–Tooth (CMT) Next-Generation Sequencing (NGS) panel from Invitae (San Francisco, CA, USA), which contained 72 CMT-related genes. The presence and absence of variants were confirmed by Sanger sequencing in all of the studied affected and unaffected family members. Variants were interpreted with strict ACMG criteria and segregation of identified variants within the family was as previously described [20]. Patients signed a consent for publication of their photos, and the study was carried out in accordance with the tenants outlined in the Declarations of Helsinki.

### 2.2. Sample Collection and Lipidomic Analysis

Sphingolipids were extracted from the collected plasma of affected and unaffected members of the family as well as two healthy controls outside the family [20]. In brief, sphingolipid bases and their phosphates were analyzed using a Prominence XR system (Shimadzu; Kyoto, Japan) with Targa C8 columns (2.1 × 20 mm, Higgins Analytical; Mountain View, CA, USA) [21]. The high-performance liquid chromatography eluate was analyzed by Multiple Reaction Monitoring on a QTRAP5500 mass analyzer in positive ion mode. The samples were analyzed for sphingolipids using precursor ion scans with internal standards, allowing for normalization and quantitation of ceramides, deoxysphingolipids, and deoxymethylsphingolipids. For graphical visualization, plasma levels were normalized for each molecular species by dividing the value of that species in each individual by the average value for that species across all participants, including controls. Absolute amounts of sphingolipid species as an average of 2–3 mass spectrometry runs per species can be found in Supplementary Table S1.

### 2.3. Literature Review

All articles from PubMed that contained both “HSAN1” and “macular telangiectasia”, “MacTel”, “ceramide”, “deoxyceramide”, or “deoxysphingolipid” were reviewed. Structural modeling was performed in PyMol based on the published Protein Data Bank (PDB) structure [2]. Maps of mutations were made using Illustrator for Biological Sequencing [22].

## 3. Familial Case Report

The proband, first reported as an HSAN1-only-affected patient by Saba, Li, and colleagues [20], is a 39-year-old male who first presented with lower limb weakness and fatigue at age 24, followed shortly after by paresthesia of the lower extremities. Subsequent neurological examination revealed bilateral lower extremity muscular atrophy and pes cavus, decreased ankle and wrist strength, absent pain sensation in the hands and lower legs, decreased vibration sense in the hands and feet, and absent patellar and Achilles reflexes.

**Figure 3:**
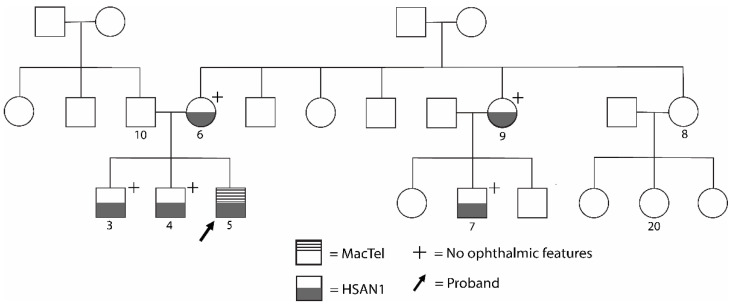
Pattern of inheritance of HSAN1 and MacTel2 in the proband’s family. Pedigree describing the phenotypes of HSAN1 and MacTel2 in the proband (individual 5) and his extended family. The proband’s brothers (individuals 3 and 4), mother (individual 6), maternal aunt (individual 9), and cousin on maternal side (individual 7) are affected by HSAN1 per physical exam but not MacTel2 (confirmed by exam and ophthalmic imaging for individuals 3 and 4, by patient report for patients 6, 7, and 9). The proband is the only participant affected by MacTel2.

The proband is the oldest of three brothers who presented with similar but less severe symptoms in their 20s and were 36 and 30 years old at the time of ocular examination (Figure 3). Both of the proband’s brothers had neurologic exams that were positive for sensory loss, ankle weakness, and hand weakness, without paresthesia or foot deformities. The proband’s mother, now 66 years old, is also affected by HSAN1. Her presentation was positive for sensory loss and pain, and she reported the onset of symptoms around age 56, which is delayed in comparison to her sons. The proband’s extended family consists of two additional relatives diagnosed with HSAN1 but without visual problems, all of whom demonstrated neurologic symptoms and age-of-onset within the range defined by the proband’s mother and two brothers.

**Figure 4:**
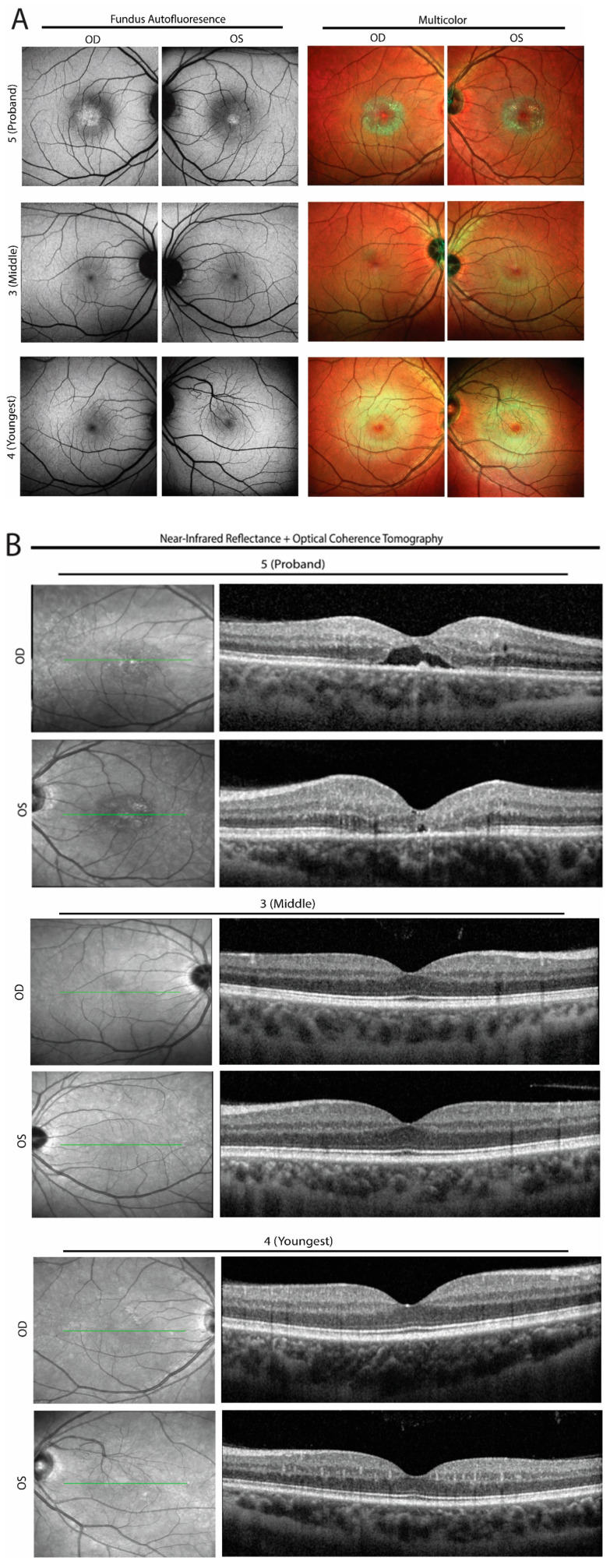
Ophthalmic imaging of the proband and his brothers. Fundus autofluorescence (FAF), multicolor, and optical coherence tomography (OCT) were obtained on the proband and his brothers (individuals 3 and 4). (**A**) FAF of the proband (individual 5, top row) demonstrates abnormal hyperautofluorescence at the fovea and surrounding parafovea, with corresponding near-circumferential deposition of crystals in the parafovea, as visualized by multicolor imaging. The middle and youngest brothers (middle and bottom rows) were normal on FAF and multicolor imaging, save for an incidentally detected congenital macrovessel in the left eye of the youngest brother, a well-described, rare condition not related to HSAN1 or MacTel2. (**B**) OCT imaging of the proband shows outer retinal loss and cavitation as well as some hyperreflective foci, especially in the right eye. OCT imaging of the brothers (middle and bottom rows) were normal. OD = right eye, OS = left eye.

The proband noticed difficulty with detailed central vision beginning at approximately age 32, eight years after the onset of his neurologic symptoms. Central vision loss progressed gradually over the ensuing years. At presentation to us at age 39, his best-corrected visual acuity was 20/60 in the right eye and 20/40 in the left eye. His pupil reactivity, intraocular pressure, and anterior segment exam were all unremarkable. His funduscopic exam and multimodal retinal imaging (including 30-degree multicolor, short-wavelength autofluorescence imaging and spectral domain optical coherence tomography (Heidelberg Spectralis OCT, Heidelberg Engineering, Franklin, MA, USA)), revealed crystalline deposits, a loss of retinal transparency, and a loss of the outer nuclear layer (ONL) in the fovea (which contains photoreceptors and specialized Müller glia). There was notable symmetry in the pathology between the two eyes (Figure 4). Compared to sporadic MacTel2, where pathology tends to occur first in the temporal parafovea, the crystalline deposits and ONL loss in the proband were highly symmetric around the fovea. A comprehensive analysis including multimodal retinal imaging of the two brothers revealed no signs of MacTel2 (Figure 4). Per the proband’s report, there are no known difficulties with central visual acuity in any other HSAN1-affected or unaffected members of the proband’s extended family.

A focused Charcot–Marie–Tooth Invitae genetic testing panel for the proband previously revealed a single pathogenic variant in *SPTLC2* c.529A>G p.(Asn177Asp) [23] that was distinguishable in all HSAN1-affected family members. Additional variants of unknown significance in *PMP22* c.130A>T p.(Thr44Ser) and *SCN11A* c.2174G>A p.(Arg725His) were identified in the proband but did not associate with disease and were not thought to be contributory [20]. Asn177 of SPTLC2 (Figure 2, magenta) is located in close proximity to the PLP cofactor and active site of the SPT complex. The causative mutation maps in close 3D proximity to the previously reported pathogenic mutations in SPTLC2 implicated in HSAN1/MacTel2 overlap, as demonstrated by molecular modeling (Figure 2A–C). Outside of the variant carried by our proband, all other HSAN1-causative point mutations identified in *SPTLC2*—p.(Arg145Ser), p.(Ala182Pro), p.(Arg183Trp), p.(Val359Met), p.(Gly382Val), p.(Ser384Phe), p.(Gly431Asp), and p.(Ile504Phe)—map closely in 3D space near the active site despite being distributed throughout the protein (Figure 2D).

**Figure 5:**
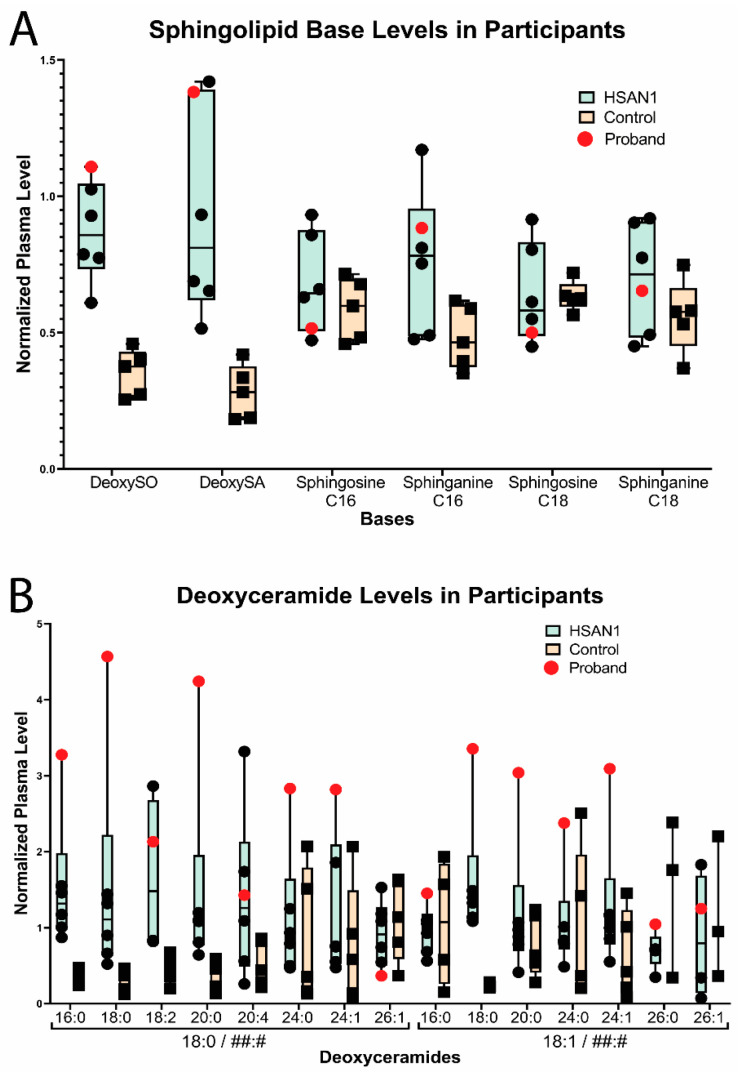

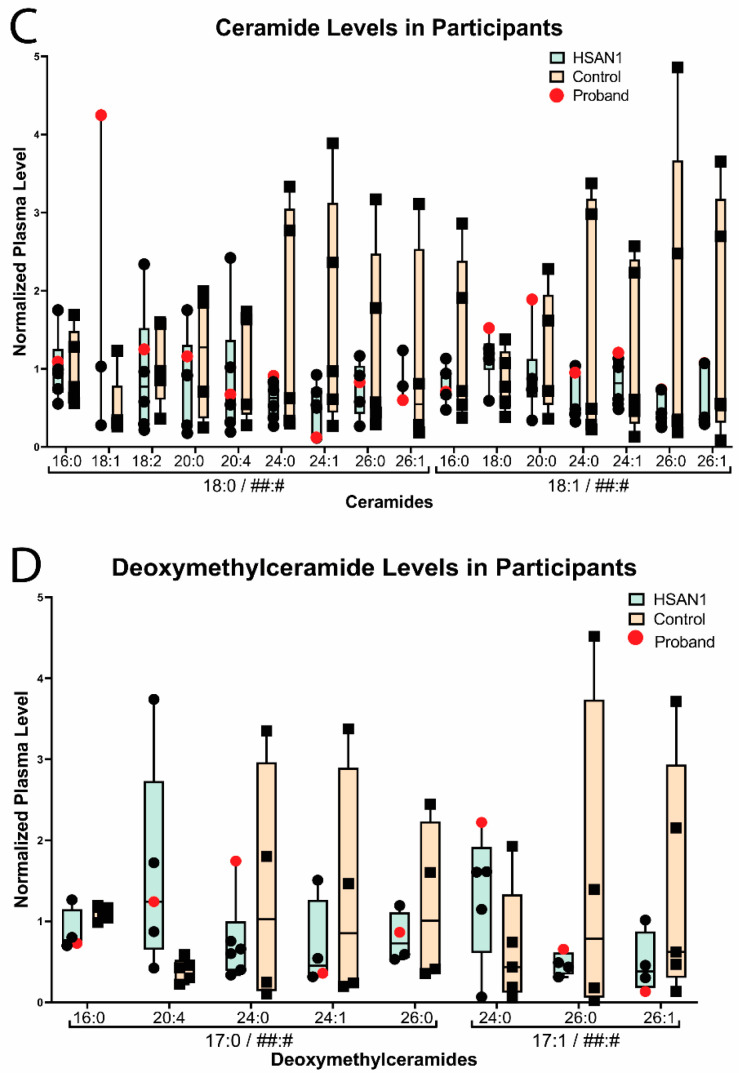
Sphingolipid bases, deoxyceramide, ceramide, and deoxymethylceramide plasma levels. Normalized plasma levels in participants with HSAN1 vs. control group measured by quantitative mass spectroscopy. Non-normalized values can be found in Supplementary Table S1. In all graphs, the proband’s value is marked in red. The nomenclature for the ceramide/deoxyceramide species, which are composed of a sphingolipid/deoxysphingolipid base plus a fatty acid, is as follows: for species (A:B/X:Y), A is the carbon length of the sphingolipid/deoxysphingolipid base, B is the number of unsaturated bonds in sphingolipid/deoxysphingolipid base A, X is the carbon length of the fatty acid, and Y is the number of unsaturated bonds in fatty acid X. The species are grouped by sphingolipid/deoxysphingolipid base (indicated by brackets, with the fatty acid placeholder denoted as “##:#”) and are labeled along the *x*-axis by their fatty acid chain. (**A**) Relative levels of deoxysphingolipid and sphingolipid bases: deoxysphingosine (deoxySO), deoxysphinganine (deoxySA), and four species of sphingolipid bases. (**B**) Relative deoxyceramide species levels, demonstrating elevated levels in the proband compared to other participants, including HSAN1-only-affected family members. This is particularly true for deoxysphingosine base (18:0) and acyl chains that were 18–20 carbons long. (**C**) Relative ceramide species levels, showing no correlation to HSAN1 status. (**D**) Relative deoxymethylceramide species levels, which also show no correlation to HSAN1 status.

Plasma sphingolipid analysis of the proband and select relatives demonstrated that levels of the deoxysphingoid bases, 1-deoxysphingosine (deoxySO) and deoxysphinganine (deoxySA), were notably higher in those affected by HSAN1 than individuals who did not have HSAN1 (Figure 5A and Supplementary Table S1). However, within the HSAN1 group, deoxySO and deoxySA levels did not strongly correlate with the proband’s HSAN1/MacTel2 overlap syndrome. In contrast, several species of deoxyceramides were markedly elevated in the proband compared to both unaffected family members and family members with HSAN1 but no MacTel2 (Figure 5B). In particular, the deoxyceramides carrying the saturated deoxysphinganine base and deoxyceramides with shorter acyl chains (especially those with 18 or 20 carbons) were highest in the proband compared to all other family members. Levels of deoxymethylceramides and ceramides did not correlate with HSAN1 or HSAN1/MacTel2 overlap (Figure 5C,D).

## 4. Discussion

The manifestation of MacTel2 in HSAN1 patients with mutations in *SPTLC1* or *SPTLC2* has opened up new insights into the pathogenesis of sporadic MacTel2, pointing towards altered sphingolipid biology as a possible pathoetiologic mechanism. However, many HSAN1 patients with pathologic *SPTLC1/2* variants do not develop MacTel2 [12]. The variable penetrance of MacTel2 among HSAN1 carriers provides an opportunity to biochemically assay sphingolipid species across HSAN1-only and HSAN1/MacTel2-overlap patients within a single family to determine which species most correlate to the overlap syndrome and therefore may drive the disease. In this report, we present an extended HSAN1 family with a *SPTLC2* variant that has never been reported in association with MacTel2 before, where only one member is affected with MacTel2, but many members, including the proband’s brothers, have HSAN1. We collect extended sphingolipid data, including ceramides, deoxyceramides, and deoxymethylceramides, on the proband (HSAN1/MacTel2 overlap), HSAN1-only-affected family members, and family members not carrying the mutation. These data hint that variable penetrance of MacTel2 among HSAN1 carriers may correlate specifically with levels of deoxyceramides with a saturated deoxysphinganine base and shorter acyl chain lengths (18 or 20 carbons). These results suggest that specific deoxyceramide levels should be investigated in sporadic MacTel2 patients as a potential biomarker of age-of-onset and disease severity.

This report also adds to the HSAN1/MacTel2-overlap literature by providing high-resolution multimodal retinal imaging of the affected proband. In contrast to sporadic MacTel2, where age-of-onset is typically in the sixth or seventh decade of life [24], the proband had symptoms starting at age 32. This early onset of MacTel2 is consistent with the reports of early MacTel2 in other HSAN1/MacTel2-overlap patients [11]. Moreover, in contrast to sporadic MacTel2, where pathology is concentrated in the temporal parafovea, our patient had symmetric parafoveal degeneration. This symmetry, combined with the high abundance of retinal crystals, subtly sets the proband’s pathology apart from typical MacTel2. The only other case of HSAN1/MacTel2 overlap from a *SPTLC2* mutation (p.Ser384Phe) involved a 52-year-old male with a 20-year history of distal to proximal sensory disturbance and sharp pains along with a 2–3 year history of lower-limb anesthesia and weakness. Retinal imaging from that case is limited to color photos, which demonstrate a fairly symmetric pigment clump centered around the fovea, potentially consistent with our observation that MacTel2 in HSAN1 patients has less of a temporal parafoveal predominance compared to sporadic MacTel2 [13]. Interestingly, retinal toxicity from tamoxifen manifest similarly to MacTel2, with a primary Müller glia degeneration [25]. Tamoxifen toxicity, however, has a more symmetric foveal involvement than sporadic MacTel2 [26]. In this way, tamoxifen toxicity loosely resembles the HSAN1/MacTel2-overlap phenotype of our proband. The mechanism of Müller cell degeneration in tamoxifen retinal toxicity is not fully understood. In the context of breast cancer cell lines, decreasing serine levels made cells more susceptible to tamoxifen toxicity while increasing serine levels had the opposite effect [27]. This observation could potentially be explained by elevated serum concentrations of toxic deoxyceramides due to a low-serine environment associated with tamoxifen, though no study has collected data on deoxyceramide levels in patients taking tamoxifen. The reasons for the symmetry of affected foveal tissue between MacTel2 in HSAN1/MacTel2 overlap syndrome and tamoxifen toxicity are unknown, but possible shared mechanisms are suggested. In contrast, the predilection of the temporal parafovea to degenerate in sporadic MacTel2 may suggest local anatomic or functional factors that lead to focal elevations of deoxyceramide levels. Alternatively, sporadic MacTel2 could be explained by higher sensitivity of the temporal fovea to deoxyceramide concentrations, and those with sporadic MacTel2 have lower levels of deoxyceramides than HSAN1 or tamoxifen toxicity patients.

All HSAN1-affected individuals in this report’s family showed increased deoxysphingolipid bases and deoxyceramide levels in comparison to unaffected individuals (Figure 5), which is consistent with previous studies showing deoxysphingolipid accumulation in HSAN1 patients [7,8]. Mutations in *SPTLC1* and *SPTLC2* have been shown to reduce the binding specificity of the SPT complex for serine, allowing for alanine or glycine binding and promoting accumulation of neurotoxic deoxysphingolipids [6,7,8,9]. The *SPTLC2* p.(Asn177Asp) variant has been found to be pathogenic for HSAN1 in two independent families, including the family in this study [20,23]. The Asn177 mutation is located within close proximity to the PLP cofactor and SPTLC2 active site, suggesting this mutation affects the binding specificity for substrates (Figure 2). It is likely that the mutation decreases specificity for serine, allowing for alanine binding in the active site and leading to the generation of deoxysphingolipids even with sufficient serine supply. Studies have found that neurons are especially sensitive to the toxic effects of deoxysphingolipids, which is evident by the relationship between deoxysphingolipids and diabetic neuropathy, despite nearly uniform expression of *SPTLC1* and *SPTLC2* across tissue types [28,29,30,31,32,33]. Indeed, lowering deoxysphingolipid plasma levels resulted in improved sensory function in a diabetic rat model [34]. Deoxysphingolipids have been shown to alter the membrane complexes of iPSC-derived neurons, leading to impaired signal transduction and the promotion of myelin breakdown [35].

While the role of deoxysphingolipids in the pathophysiology of HSAN1 is heavily supported, the underlying mechanism by which deoxysphingolipids may drive MacTel2 is not well-understood. Deoxysphingolipids are elevated in the serum of sporadic MacTel2 patients [11]. Additionally, an induced pluripotent stem cell model of MacTel2 using the most common disease variant in the serine synthesis enzyme PHGDH also triggers elevated deoxysphingolipid production [16]. One possible mechanism of toxicity may be inferred from the pathologic roles of closely related ceramide species. Ceramides are physiologic lipids that play a role in cell signaling and apoptosis. They are maintained at low intracellular levels by quick transformations into less toxic sphingolipid species [36,37]. Stress within the cell can induce ceramide accumulation in membranes [37], helping form rigid rafts that induce negative curvature, which consequently alters both membrane fluidity/permeability [38] and cell signaling, including inflammatory signaling [39]. Especially in the mitochondria, changes in permeability cause the release of apoptogenic proteins, inhibition of the electron transport chain, generation of reactive oxygen species, and depletion of ATP, all of which induce apoptosis [37,40]. Deoxyceramides cannot be metabolized by the same pathways as ceramides because they lack a C1 hydroxyl group (highlighted in red in Figure 1), which inhibits their recognition by enzymes involved in ceramide metabolism. As a result, they are considered dead-end products [7,41]. Since they are otherwise identical in structure, it is speculated that deoxyceramide accumulation has similar detrimental effects on the cell as ceramide accumulation. This is supported by a study that observed deoxydihydroceramides and deoxyceramides promoting apoptosis [42]. The possible effects of deoxyceramides on both inflammatory signaling and membrane stability would play a particularly important role in Müller glia, which help mediate retinal inflammation and which have unusually high mitochondrial demands [43].

Of all deoxyceramide species assayed in this study, the species with shorter acyl chains, particularly those with 18 or 20 carbons, correlated most strongly with the HSAN1/MacTel2-overlap phenotype. There is evidence that longer-chain ceramides may have less detrimental effects on membrane curvature and signaling and that ceramides with very-long-chain fatty acids (24 carbons and longer) may actually be critical for certain aspects of retinal function [44]. In pathologic processes ranging from insulin resistance [45] to atopic dermatitis [46], very-long-chain fatty acid ceramides are associated with protection, whereas ceramides with acyl groups in the 16–20 carbon range are associated with worsened disease. As discussed above, the biophysical properties of ceramides and deoxyceramides may be similar enough that they would have similar pathologic mechanisms, but deoxyceramides accumulate since enzymes involved in ceramide degradation do not recognize deoxyceramides. It would be interesting to know whether HSAN1 patients who do not develop MacTel2 have variants in certain fatty acid elongases that are responsible for producing very-long-chain fatty acids [47]. Such variants could help protect these individuals from the increased toxicity of deoxyceramides by skewing deoxyceramide abundance towards those less toxic deoxyceramides with very-long-chain fatty acids (24 carbons and longer).

We acknowledge significant limitations of our study. The findings that certain deoxyceramide species are higher in our proband are intriguing, but they need to be replicated in other families with variable penetrance of the HSAN1/MacTel2-overlap syndrome to be labeled as a true correlative predictor of HSAN1/MacTel2 overlap. Further studies would also be needed to prove that the high levels of certain deoxyceramide species in our proband are causative of MacTel2 rather than just correlative.

Overall, our data hint at a role for deoxyceramides, particularly those with a deoxysphinganine base and 18–20 carbon acyl chain, in the development of MacTel2 in patients with mutations in the SPT complex. Comprehensive sphingolipid profiling in larger cohorts of families with variable penetrance of HSAN1/MacTel2 overlap may solidify whether particular species of deoxyceramides are predictive markers for the onset of MacTel2 in HSAN1 patients. Any biochemical species that are shown to be strongly predictive of MacTel2 in HSAN1 patients may also contribute to our understanding of the pathogenesis of sporadic MacTel2 and tamoxifen-induced retinal degeneration.

## Data Availability

All relevant data are contained within the manuscript text and supplementary material, and any additional underlying data are available upon request.

## Supplementary Materials

The following supporting information can be downloaded at: https://www.mdpi.com/article/10.3390/genes14040931/s1, Supplementary Table S1: Plasma sphingolipid bases, ceramides, deoxyceramides, and deoxymethylceramides in participants with and without HSAN1.
