# Supplementary material for: Specific Deoxyceramide Species Correlate with Expression of Macular Telangiectasia Type 2 (MacTel2) in a SPTLC2 Carrier HSAN1 Family
Source: Genes (Basel). 2023 Apr 18;14(4):931. doi: 10.3390/genes14040931 (PMC10137565; doi:10.3390/genes14040931)
Supplement: Supplementary file 1 [file genes-14-00931-s001.zip › genes-2246099-supplementary.pdf]

	HSAN1 Affected						Control				
**Species**	**3**	**4**	**5**	**6**	**7**	**9**	**8**	**10**	**13***	**14***	**20**
Deoxysphingosine	0.895	1.156	1.628	1.507	1.137	1.365	0.591	0.674	0.402	0.553	0.374
Deoxysphinganine	0.463	0.661	0.980	1.007	0.365	0.488	0.200	0.297	0.133	0.237	0.130
Sphingosine (C16)	13.041	18.225	14.254	25.761	17.399	23.715	16.512	12.667	18.723	13.332	19.743
Sphinganine (C16)	0.206	0.491	0.340	0.316	0.199	0.370	0.165	0.147	0.247	0.194	0.259
Sphingosine (C18)	49.234	88.283	54.883	100.425	60.368	67.224	78.972	61.986	69.713	68.429	68.647
Sphinganine (C18)	1.749	3.569	2.538	3.007	1.912	3.508	2.064	1.436	2.240	2.252	2.904
Cer(18:0/16:0)	5.093	6.464	7.487	12.019	3.804	6.806	5.269	4.323	8.781	3.855	11.593
Cer(d18:0/18:1)			3.471		0.227	0.841	0.248	1.006	0.215	0.251	0.279
Cer(d18:0/18:2)	2.992	2.221	12.749	23.850	5.905	9.829	16.044	3.679	8.682	9.933	16.294
Cer(d18:0/20:0)	2.106	3.996	2.644	2.088	0.405	0.631	1.613		4.216	0.572	4.547
Cer(d18:0/20:4)	32.122	52.827	112.457	405.247	90.668	170.728	276.671	46.266	91.534	289.709	273.314
Cer(d18:0/24:0)	26.787	72.422	91.213	83.273	37.345	52.696	62.610	30.031	333.596	33.700	277.329
Cer(d18:0/24:1)	4.685	1.006	1.051	6.117	4.364	8.047	8.466	5.311	20.592	2.342	33.905
Cer(d18:0/26:0)		1.967	1.397	1.541	0.448	0.975	0.963	0.747	5.347	0.488	3.001
Cer(d18:0/26:1)		4.758	2.302	2.994			0.713	1.092	11.973		3.108
Cer(d18:1/16:0)	142.966	197.111	148.214	140.538	99.752	237.357	113.833	149.953	400.105	78.515	600.735
Cer(d18:1/18:0)	27.206	55.120	70.070	57.970	52.001	51.332	35.554	26.743	63.423	17.712	49.424
Cer(d18:1/20:0)	31.235	80.067	173.191	68.432	67.215	67.406	65.584	65.633	209.015	32.943	148.538
Cer(d18:1/24:0)	1050.920	2629.447	2407.073	1226.999	818.531	1099.352	1233.406	721.930	8545.707	567.155	7544.318
Cer(d18:1/24:1)	491.805	1015.916	1083.175	548.923	432.859	916.241	542.285	412.446	2000.564	116.709	2305.476
Cer(d18:1/26:0)	21.748	43.144	42.948	25.336	15.638	14.755	20.509	19.299	284.518	11.060	145.068
Cer(d18:1/26:1)	6.805	20.763	20.721	5.635	6.020	7.334	10.748	10.303	70.677	1.690	52.195
Cer(m18:0/16:0)	9.483	6.164	20.040	8.928	5.320	7.177	1.495	2.891	2.197	2.134	1.464
Cer(m18:0/18:0)	16.235	41.071	142.472	44.875	20.641	28.035	6.137	14.530	13.656	3.814	11.551
Cer(m18:0/18:2)			12.662	17.011	4.859	4.936	2.107	3.705	1.159	3.034	4.003
Cer(m18:0/20:0)	38.983	43.080	152.792	39.561	23.054	29.071	8.277	16.535	21.327	4.847	18.540
Cer(m18:0/20:4)	1.870	4.040	10.310	23.949	7.865	12.550	2.016	3.168	1.544	6.181	5.920

Cer(m18:0/24:0)	85.609	135.384	306.970	101.169	51.039	54.180	17.973	38.105	224.332	14.038	163.879
Cer(m18:0/24:1)	52.406	128.464	194.975	51.788	32.646	38.245	9.679	40.300	142.961	6.172	63.627
Cer(m18:0/26:1)	37.785	17.393	11.694	48.834	23.669	34.651	51.434	25.768	11.750	52.194	36.373
Cer(m18:1/16:0)	2.194	1.800	4.661	3.520	3.377	2.973	1.855		5.034	0.479	6.203
Cer(m18:1/18:0)	18.442	22.530	57.052	23.749	19.336	25.301	3.840	4.576	4.835	3.509	3.962
Cer(m18:1/20:0)	32.004	68.210	235.888	75.381	60.447	82.984	41.612	49.461	89.906	21.606	96.061
Cer(m18:1/24:0)	52.465	109.294	257.327	87.631	85.385	90.076	22.363	39.614	271.229	21.939	153.564
Cer(m18:1/24:1)	39.078	83.292	219.579	60.617	70.815	81.782	15.115	29.706	103.155	6.434	71.730
Cer(m18:1/26:0)		3.738	5.440	3.696	3.629	1.806	1.777		12.417		9.161
Cer(m18:1/26:1)		4.645	3.174	0.177		0.863		0.921	5.593		2.408
Cer(dm17:0/16:0)			1.099	1.911	1.056	1.209	1.768	1.570	1.488	1.685	1.804
Cer(dm17:0/20:4)		1.698	4.998	15.049	3.513	6.932	1.218	1.716	0.902	1.844	2.372
Cer(dm17:0/24:0)	12.386	10.342	53.690	18.495	20.267	23.219	7.675		103.105	3.172	55.444
Cer(dm17:0/24:1)			0.308	1.297	0.271	0.467	0.207		2.903	0.168	1.261
Cer(dm17:0/26:0)			1.690	2.332	1.157	1.037	0.696		4.774	0.807	3.131
Cer(dm17:1/24:0)		1.467	48.322	34.958	24.967	35.069	9.467	1.174	41.909	4.038	16.166
Cer(dm17:1/26:0)			3.347	2.497	1.594	2.223	0.910		23.056	0.092	7.107
Cer(dm17:1/26:1)			0.572	4.333	1.948	1.296	2.649	0.574	15.864	2.003	9.197

Table S1. Plasma Sphingolipid bases, Ceramides, Deoxyceramides, and Deoxymethylceramides in participants with and without HSAN1. Ceramide species are denoted “Cer(d##/##)”, Deoxyceramides are denoted “Cer(m##/##)”, and Deoxymethylceramides are denoted by “Cer(dm##/##)”.

* Control participants that are NOT members of this family
